# Integrative Multiomics Evaluation of IIDH1 Metabolic Enzyme as a Candidate Oncogene That is Correlated with Poor Prognosis and Immune Infiltration in Prostate Adenocarcinoma

**DOI:** 10.1155/2022/9854788

**Published:** 2022-01-29

**Authors:** Chen-Yueh Wen, Kuan-Hao Tsui, Chiung-Hung Chang, Yi-Han Chiu, Shu-Chuan Amy Lin, Ching-Yu Chu, Chia-Jung Li

**Affiliations:** ^1^Division of Urology, Department of Surgery, Kaohsiung Veterans General Hospital, Kaohsiung 813, Taiwan; ^2^Department of Obstetrics and Gynecology, Kaohsiung Veterans General Hospital, Kaohsiung 813, Taiwan; ^3^Institute of Biopharmaceutical Sciences, National Sun Yat-Sen University, Kaohsiung 804, Taiwan; ^4^Department of Traditional Chinese Medicine, Tainan Municipal Hospital, Tainan 701, Taiwan; ^5^Department of Microbiology, Soochow University, Taipei 111, Taiwan; ^6^Department of Nursing, National Yang Ming Chiao Tung University Hospital, Taipei 112, Taiwan; ^7^Nursing School of National Yang Ming Chiao Tung University, Yilan 260, Taiwan

## Abstract

Mutations in the isocitrate dehydrogenase gene (IDH1) are involved in the progression of tumors. Although IDH1 has a role in various tumors, its clinical relevance and its expression in response to the immune response have not been investigated in prostate adenocarcinoma (PRAD). In the present study, we investigated the utility of IDH1 as a prognostic biomarker for PRAD by analyzing IDH1 mRNA expression and its association with patient survival and immune cell infiltration. IDH1 mRNA expression was significantly higher in PRAD tissue than in normal tissue, and Kaplan–Meier survival analysis showed that IDH1 expression was significantly associated with poor prognosis in PRAD patients. To elucidate the mechanisms involved, the correlation between IDH1 expression and the level of immune cell infiltration, in particular of immunosuppressive cells such as CD8+ T-cells, CD4+ T-cells, and macrophages, was further analyzed by single-cell RNA sequencing. We also screened a pharmacogenetic database for IDH1-specific drugs that inhibited high expression in PRAD. In the present study, we used a combination of databases to identify a significant correlation between IDH1 expression and cellular infiltration and to explain the mechanism by which IDH1 confers poor prognosis in PRAD, thus demonstrating the relevance of IDH1 expression as a prognostic biomarker with clinical utility in PRAD patients.

## 1. Introduction

Prostate adenocarcinoma (PRAD) is the most common cancer in men, and approximately 250,000 new cases of PRAD are diagnosed each year in America. PRAD is characterized by a high mortality rate and was the second leading cause of cancer-related deaths among men in 2021 [1]. Although PRAD is highly prevalent, relatively little is known about its etiology. The possible risk factors of PRAD include endogenous elements, such as heredity, race, and hormone levels, and exogenous elements, such as diet, environment, and occupation [2, 3]. Determination of the genetic basis of PRAD has been challenging, and the identification of novel molecular biomarkers that could predict disease prognosis has been a research focus. The identification of such biomarkers will also contribute to understanding of PRAD pathogenesis and provide precision management for patients with PRAD.

Cytosolic isocitrate dehydrogenase 1 (IDH1) is an NADP + -dependent enzyme that metabolizes isocitrate to *α*-ketoglutarate (KG). IDH1 mutations have recently been discovered in nearly 80% of gliomas or glioblastomas and nearly 20% of acute myeloid leukemias, eliciting new interest in defining IDH1 functions *in vivo* [4–6]. IDH1 mutations are an important factor in early carcinogenesis. The R132 zone of IDH1 has neomorphic enzymatic activity, catalyzing the NADPH-dependent reduction of *α*-KG to R(-) 2-hydroxyglutarate (HG) [7, 8]. Although the role of IDH1 in several tumors has been identified, the expression patterns of IDH1 and its relationship with clinicopathological characteristics and prognosis have not been fully reported in PRAD. To understand the mechanisms of IDH1 and immune cell interactions in PRAD, in the present study, we analyzed a PRAD cohort from a multiomics database.

With the rapid development of precision medicine techniques and the establishment of various public databases, a comprehensive analysis of IDH1 has become possible [9]. A small proportion of PRAD patients respond to immunotherapy, meriting further investigation [10]. In the present study, we performed a comprehensive bioinformatics analysis of IDH1 expression in PRAD patients and evaluated its potential value as a prognostic factor for PRAD. Our results provide new directions for improving prognostic accuracy for PRAD and highlight the relevance of IDH1 in PRAD. We identified new immune pathways that could be used to stratify PRAD patients into favorable and unfavorable risk groups for responding to current immunotherapy strategies. We further explored the relationship between metabolic alterations, immune cell tumor infiltration, immunotherapy candidates, and precision therapy.

## 2. Results

### 2.1. Characteristics, Mutations, and Copy Number Changes in PRAD

**Figure 1:**
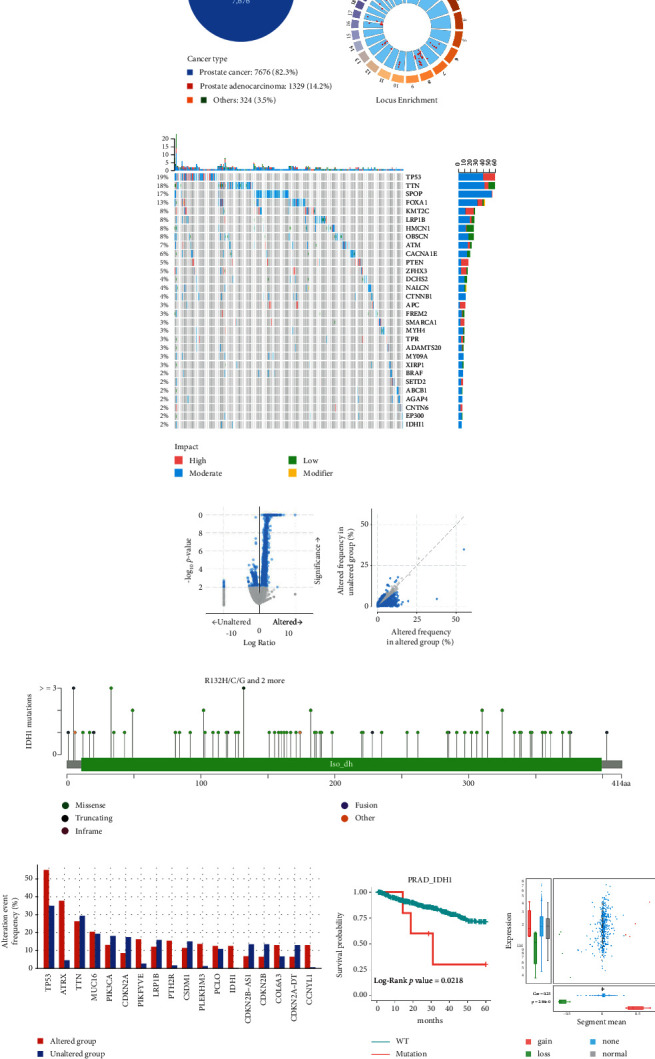
Comprehensive gene expression analysis associated with IDH1 mutations in PRAD. (a) Percentage of prostate cancer types in cBioPortal data set of 9329 patients. (b) The locus enrichment map uses red dots to mark the motifs on each chromosome in PRAD. (c) Waterfall plot of the top 30 mutated genes in PRAD, with bars indicating the proportion of mutations per patient. (d and e) Volcano and scatter plots showing the frequency of mutation-associated gene changes in IDH1 in PRAD. (f) IDH1 protein structure showing the location of specific mutations. (g) Box marks of the most frequently altered genes in PRAD. (h) Association between IDH1 mutations and survival in PRAD. (i) Distribution and correlation of CNV in prostate cancer are marked in three color to visualize the distribution of CNV ratios.

The TCGA database of prostate cancer patients (*n* = 9329) was analyzed to identify changes in prostate cancer genes and to screen for potential biomarkers. The majority of prostate cancer malignancies in the database are attributed to prostate cancer (82.3%) and prostate adenocarcinoma (14.2%) (Figure 1(a)). The motif enrichment map uses red dots to mark the motifs on each chromosome. Each red dot represents a gene and its associated position on the chromosome, and the value represents the correlation between RNA expression and copy number variation (CNV) (Figure 1(b)). We next analyzed the relationship between the top 30 mutation-driven genes and PRAD (Figure 1(c)). IDH1, which is involved in metabolic regulation, has a 2% mutation rate in PRAD patients in the database. Therefore, we further analyzed IDH1 mutations using the TCGA database to determine how these mutations may regulate gene expression and generated volcano plots and scatter plots to illustrate this (Figures 1(d) and 1(e)). We listed several of the most frequently altered genes in the data set, and the frequency of IDH1 gene mutations was high in PRAD patients Figure 1(f)), while the survival rate of patients with IDH1 mutations was low. However, based on the CNV ratio distribution and box plots, IDH1 alterations are not significant for gain or loss of function.

### 2.2. Mutational Load and Gene Expression of IDH Family Genes

**Figure 2:**
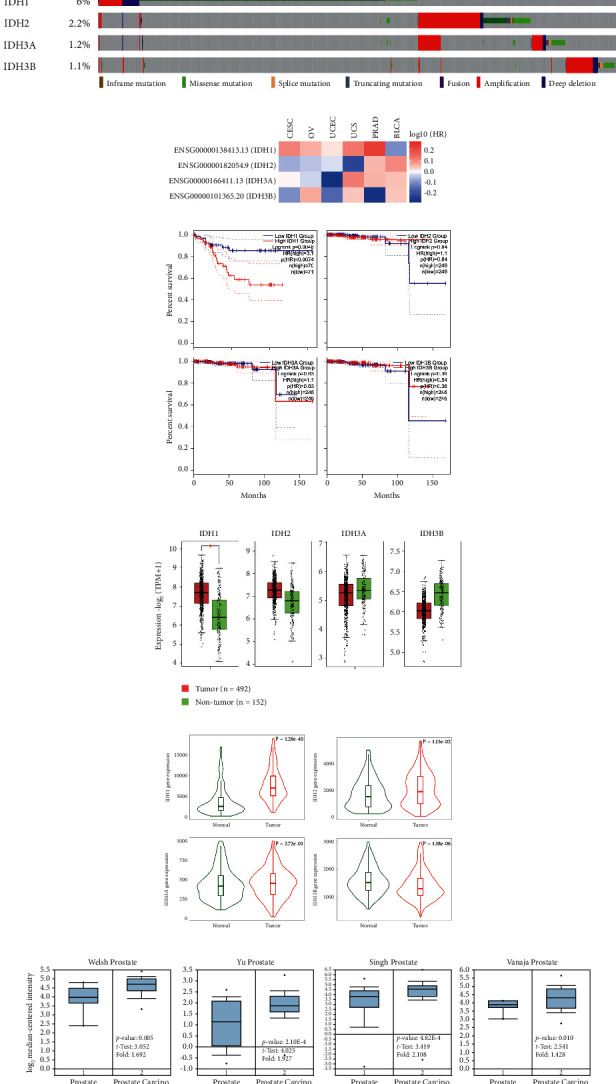
Levels of IDH family genes in PRAD. (a) Distribution and proportion of mutations in IDH family genes in the cBioportal database. (b) Heat map of the survival rate of IDH family genes in the human reproductive system. (c) Expression and survival of IDH family genes in PRAD. (d) Transcriptional levels of IDH family genes in PRAD. (e) Violin plots showing the mRNA expression levels of IDH family genes in PRAD. (f) Box and whiskers plots of oncomine data on IDH1. ^*∗*^*p* < 0.05.

**Figure 3:**
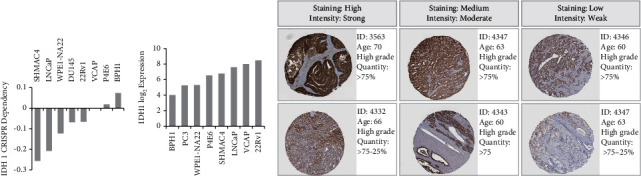
Levels of IDH1 expression in PRAD. Relative expression of IDH1in PRAD cells and tissues based on multiple databases. (a) Distribution of IDH1 CERES scores (*y*-axis) in prostate cancer cell lines. Six of 8 cell lines screened in CRISPR cell lines have a greater than 75% probability of being dependent on IDH1. (b) The mRNA detects the expression level of different prostate cancer cells. (c) Comparison of immunohistochemical images of IDH1 in PRAD tissues of six different patients based on human protein atlas.

The cBioPortal tool was applied to explore genetic alterations in IDH1-containing genes and their correlation with overall survival (OS) in PRAD patients. We investigated whether genes with *a* >6.6% or higher mutation frequency in PRAD patients were similarly altered in IDH family genes, or were altered in a more general way in other cancer types (Figure 2(a)). The TCGA and GEPIA2 data sets were used to compare IDH family gene expression and prognosis in patients with different reproductive tumor types. IDH1 is highly expressed in PRAD patients in the reproductive system (Figure 2(b)) and is associated with decreased survival (Figure 2(c)). Among 492 PRAD patients, only IDH1 transcript levels were higher in the tumor tissue than in the nontumor tissue (Figure 2(d)). Similarly, IDH1 gene expression was also higher in tumors than in matched nontumor tissue (Figure 2(e)). Overall, the analysis of this data set revealed that IDH1 upregulation is associated with prostate cancer and plays an important role in tumor progression (Figure 2(f)). We also found a significant enrichment of IDH1 dependence in prostate cancer cell lines in the CRISPR-Cas9 data set. Specifically, among prostate cancer cell lines, six out of eight (75%) prostate cancer cell lines were dependent on IDH1 (Figure 3(a)). We confirmed that IDH1 mRNA levels were significantly higher in prostate cancer cell lines, in accordance with the other data sets (Figure 3(b)). In addition, immunohistochemistry of pathological sections from the Human Protein Atlas (HPA) revealed that the protein expression of IDH1 was elevated in the TMA of PRAD patients (Figure 3(c)).

### 2.3. Functional Enrichment Analysis of IDH1 and Coexpressed Genes in PRAD

**Figure 4:**
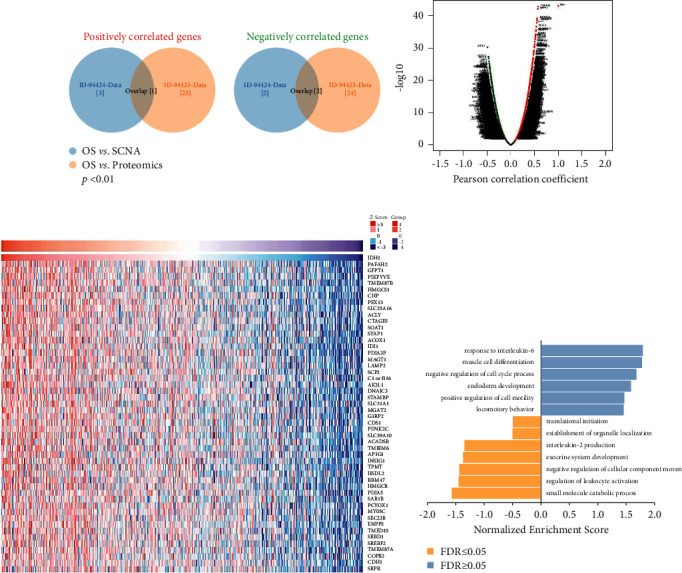
Comparisons of gene expression profiles in IDH1 and enrichment pathway analysis. (a) The Venn diagram analysis with directional constraint identified three overlapping genes between the two platforms. (b) Analysis of differential gene expression associated with IDH1 in PRAD using the Pearson test. (c) Heatmap showing the top 50 genes are each significantly positively associated with IDH1. (d) Functional enrichment analysis of IDH1 in PRAD.

To further explore the potential functions and molecular pathways of IDH1-associated genes in PRAD, we identified IDH1 coexpressed genes in 497 patients from the TCGA data set using the LinkedOmics database. A total of 20051 IDH1-associated genes were dysregulated, reflecting the important impact of the core gene IDH1 on the pathogenesis of PRAD. These clusters of genes positively associated with IDH1 are shown as red dots, while clusters of genes negatively associated with IDH1 are indicated by green dots in the volcano plot (*p* < 0.01, FDR <0.01, Figure 4(a)).  Figure 4(b) shows the top 50 genes that are significantly positively associated with IDH1 (Figure 4(c)). These IDH1-associated coexpressed genes are mainly involved in interleukin-6, muscle cell differentiation, negative regulation of cell cycle processes, endoderm development, positive regulation of cell motility, locomotory behavior, translational initiation, establishment of organelle localization, interleukin-2 production, exocrine system development, negative regulation of cellular component movement, regulation of leukocyte activation, and small-molecule catabolic processes (Figure 4(d)).

### 2.4. Correlation between IDH1 Expression and Infiltrating Immune Cells

**Figure 5:**
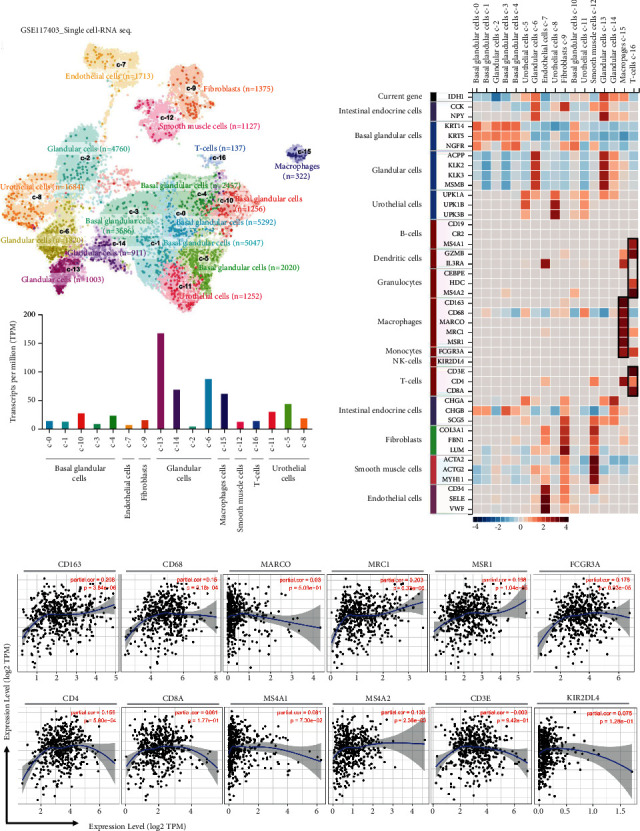
The correlation between IDH1 and immunization. (a) Single-cell-RNA sequencing in identified single-cell-type clusters in prostate cells as shown by UMAP plots and bar graphs. (b) Heat map showing the expression of IDH1 gene and well-known cell-type markers in different single-cell-type clusters of the tissue. The left panel shows which cell type each marker is associated with. (c) Relationship between immune cell infiltration and IDH1 expression from the timer database.

The single-cell RNA sequencing data set was based on a meta-analysis of single-cell RNA sequencing literature and a single-cell databases including healthy human tissue. We used this database (10x Genomics) to analyze the potential associations between IDH1 and the tumor microenvironment in prostate cancer. This single-cell RNA sequencing data set included studies with >4,000 cells and 20 million reads and included a data set whose pseudo-batch transcriptome expression profiles were highly correlated with the transcriptome expression profiles of HPA tissue batch samples. We extracted transcriptomes from 35,862 single cells and 16 cell type clusters as UMAP plots and bar charts (Figure 5(a)). These cells were analyzed for IDH1 specificity and distribution to determine the differences in the number of genes in these single-cell types and the number of genes detected in all cell types. The heat map in Figure 5(b) shows the expression of IDH1 and previously characterized cell-type markers in the different single-cell type clusters. We found a correlation between IDH1 and immune cells based on single-cell sequencing results (Figure 5(c)). Therefore, we further analyzed the genes that were associated with IDH1 immunity in the available data sets.

**Figure 6:**
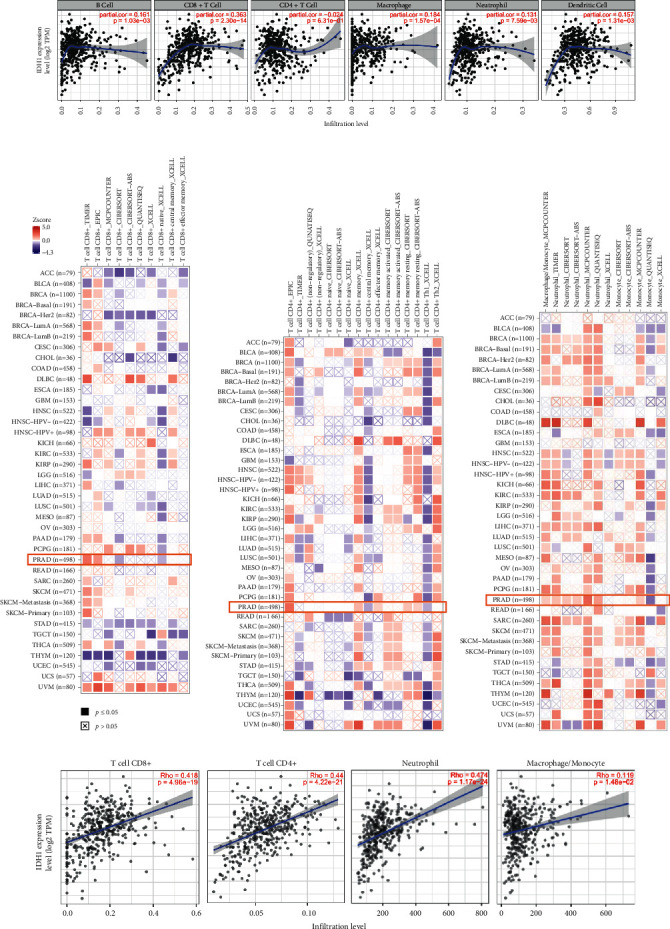
IDH1 is closely related to immunity in PRAD. (a) TIMER analysis of the correlation between IDH1 expression in PRAD and purity correction among six immune cells. (b–d) Correlation analysis of IDH1 gene expression with T-cell CD8+, T-cell CD4+, macrophage, and monocyte immune infiltration. (e) Positive correlation between IDH1 levels and infiltration levels of CD8+ T cells, CD4+ T cells, macrophages, and neutrophils in PRAD from TIMER database.

We further evaluated the relationship between IDH1 and various tumor-infiltrating immune cells. The TIMER data showed that IDH1 was positively associated with CD163, CD68, MRC1, MSR1, and CD4. We hypothesized that IDH1 could regulate the immune response, and we therefore further extended our analysis to analyze the correlation between IDH1 and the level of infiltration of different immune cells (Figure 6(a)). Notably, there was a high positive correlation between IDH1 expression and the level of CD8+ and CD4+ T cell infiltration. We comprehensively screened IDH1 profiles for all tumors and used seven algorithms to analyze the expression in each cancer type and correlated this with IDH1 expression levels (Figures 6(b)−6(d)). IDH1 is highly correlated with CD8+ T cells (rho = 0.418), CD4+ T cells (rho = 0.44), and neutrophils (rho = 0.474), while macrophages/monocytes (rho = 0.119)) were not correlated (Figure 6(e)).

### 2.5. Screening for Drugs That Potentially Inhibit IDH1 by Pharmacogenomics

**Figure 7:**
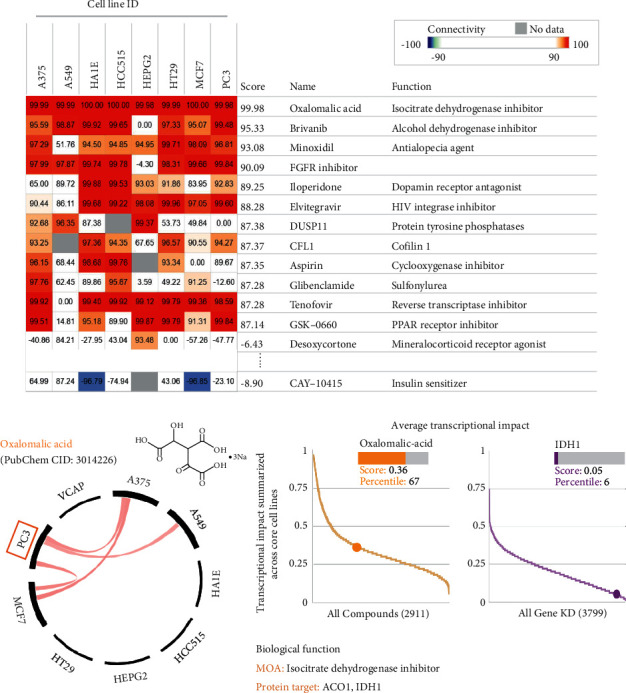
*In silico* and *in vitro* characterization of the inhibitory activity of oxalomalic acid against IDH1 in prostate cancer cells. (a) Oxalomalic acid treatment simulates the effect of IDH1 inhibition on prostate cancer cell lines. (b) CMap analysis was performed to explore the similarity between IDH1 and drug-induced genetic features in multiple cancer cell lines to assess the effect.

CMap analysis was performed to identify potential drugs that inhibit IDH1. The CMap database provides gene signatures and screens for associations between specificity and drug-driven gene expression [11]. Figure 6(a) shows the top 10 perturbagens that mimic IDH1-driven gene signatures, including an EGFR inhibitor with a score of 90.09, which also inhibits IDH1 overexpression. In comparison, CAY-40145 had a score of −8.9 (Figure 7(a)). We further retrieved the IDH1 gene library from the pharmacogenomic database to search for potential drugs for the treatment of PRAD. As shown in Figure 7(b), we found that the scores of oxalomalic acid and IDH11 knockdown against prostate cancer cells (PC3) were 0.36 and 0.15, respectively, indicating a positive correlation between the average transcriptional impact of IDH1 expression and oxalomalic acid drug activity. These results suggest that oxalomalic acid may ameliorate the IDH1-related cancer signature.

## 3. Discussion

PRAD is one of the most common tumors in older men. It is characterized by low-grade malignancy, and most PRAD tumors are located in the prostate and adjacent organs [12, 13]. The 10-year survival rate for patients with PRAD is as high as 98%, with surgery or active surveillance being the only options in most cases [14, 15]. Although most cases are localized, the number of men who first present with or progress to metastatic PRAD is now increasing. Advanced PRAD can be divided into two groups: those who present with *de novo* metastases and those who rapidly progress to advanced disease after surgery [16]. Several studies have illustrated that abnormal gene expression affects survival in patients with advanced PRAD [17, 18]. However, few studies have discussed the infiltration of immunosuppressive cells and metabolic changes in advanced PRAD. In the present study, we used bioinformatics analysis, PRAD tissue microarray data, and multiple types of PRAD cell lines to screen for mRNA expression that may be prognostically relevant to PRAD patients, providing the basis for future clinical and scientific research.

Several reports have suggested that IDH1 expression is dysregulated in various cancers [19, 20]. However, relatively few studies have discussed the relationship between IDH1 and PRAD. Hinsch et al. mentioned that only 0.3% of PRAD patients have IDH1 mutations, and that their relevance to immunity is unclear [21]. In the present study, by analyzing multiple databases, we have provided more specific data on IDH1 characterization, mutational load, gene expression, immune cell infiltration, and pathway crosstalk in PRAD. Our Kaplan–Meier analysis suggests that IDH1 and other IDH family genes may be poor prognostic biomarkers for PRAD. However, both the single-cell RNA sequencing and immune response data suggest that IDH1 is important during PRAD.

In the present study, we determined that IDH1 expression correlates with different levels of immune infiltration in PRAD. IDH1 mRNA expression levels were positively correlated with the infiltration levels of CD8+ T cells, CD4+ T cells, macrophages, and neutrophils. Furthermore, the correlation between IDH1 expression and immune cell marker genes implies a role for IDH1 in regulating PRAD tumor immunology. M2 macrophage markers, such as CD163, CD68, MRC1, and MSR1, were correlated with IDH1 expression. These results suggest a potential role of IDH1 in regulating tumor-associated macrophage (TAM) polarization. In addition, a correlation was found between IDH1 expression and several T cell markers, DCs, and neutrophils in PRAD. These correlations suggest a potential mechanism for IDH1 regulation of immune cells in PRAD. Together, these findings suggest that IDH1 plays an important role in the recruitment and regulation of immune infiltrating cells in PRAD.

Oxalomalic acid mimics the effects of IDH1 inhibition on prostate cancer cell lines and could ameliorate the pathology of prostate cancer cells. Oxalomalic acid can modulate the targeting of IDH1 because it is a competitive inhibitor of IDH by a mechanism that regulates intracellular reactive oxygen species through IDH inactivation, and thus inhibits tumor cell migration by downregulating matrix metalloproteinase-9 [22, 23]. Oxalomalic acid also downregulates iNOS expression [24]. Thus, oxalomalic acid is a potential therapeutic agent for treating PRAD progression and metastasis via IDH1 inhibition.

One of the dilemmas of PRAD is the progression of cancer to metastatic goitre-resistant prostate cancer (mCRPC). Historically, the average overall survival of patients with untreated mCRPC has been less than 2 years, based on metastatic disease and the presence of symptoms [11, 25]. With the advent of newer drugs, such as the novel anti-androgen drug Sipuleucel-T, radiopharmaceuticals, and PARP inhibitors, many studies have demonstrated an increase in median overall survival with their use early in the disease process. Due to the poor prognosis and identification of potential drug resistance mechanisms, the selection of the correct sequence and type of systemic therapy in mCRPC is important. However, our study lacked a bioinformatics analysis of mCRPC. In the future, we should focus on mCRPC cases to collect more information.

There were some limitations to the present study. First, although the analysis of the available data sets suggests that the profile of the molecular IDH family is a potential indicator of PRAD, we do not have large sample sizes and animal studies to support these results. This can be addressed in subsequent experiments. Second, the study did not conduct some surveys on treatment outcomes in PRAD patients, and further studies are needed to validate the importance of the treatment effect of IDH1. Third, because of the inconsistent data sets in the database, some conflicting data need further clarification. Finally, to improve the value of IDH1 as a biomarker, it is important to explore its prognostic role in PRAD patients. The hypotheses generated in the present study therefore support the expanded investigation of IDH1 as a prognostic biomarker for PRAD, particularly complex PRADs like mCRPC, in a larger clinical cohort.

## 4. Materials and Methods

### 4.1. GEO Database

The Gene Expression Omnibus (GEO) database is maintained by the National Center for Biotechnology Information (NCBI). In the present study, we used the keywords “prostate cancer” and “metabolism” to search the database, and the microarray data were extracted from the whole-genome RNA for all samples.

### 4.2. cBioPortal

The cBioPortal database (Memorial Sloan Kettering Cancer Center) explores the genomic signature of tumors at the DNA level, including mechanistic information [26]. Differential gene expression, survival analysis, and immune infiltration analysis can be performed at the phenotypic level. In the present study, we used this tool for survival, mutation, copy number alteration, and overall survival (OS) analysis for common DEGs.

### 4.3. GEPIA2

An updated version of the GEPIA (Gene Expression Profiling Interaction Analysis) database, GEPIA2, was used to aggregate and analyze RNA sequencing expression data from the TCGA and GTEx projects, including 9,736 tumor and 8,587 normal samples, using a standard processing flow (14). GEPIA2 was used to examine the mRNA expression of IDH1 in PRAD and its association with survival outcomes, including disease-free survival (DFS) and overall survival (OS) [27].

### 4.4. WebGestalt

The WebGestalt database (Zhang laboratory) is a multispecies database containing 354 genetic identifiers from various databases and technology platforms and 321,251 functional categories from public databases and computational analyses [28]. In the present study, we used this tool to conduct an online gene functional enrichment analysis and to perform GO analysis of key genes interacting with IDH1 genes.

### 4.5. LinkedOmics

The mRNA data sets of PRAD patients were downloaded using LinkedOmics software [29], and a total of 499 cases containing both IDH1 expression levels and complete clinical data were screened. The patients ranked in the top 50% of IDH1 expression were assigned to the ‘high expression' group and the remaining patients were assigned to the “low expression” group. A total of 549 RNA gene microarrays containing IDH1 expression levels were selected, and 20,051 genes were detected. The Pearson correlation between each gene expression level and the IDH1 expression level was performed using an online analysis tool, and the 50 genes with a positive correlation to IDH1 expression and the highest correlation coefficient were selected for gene heat map analysis.

### 4.6. TIMER

To systematically analyze the immune cell infiltration in different cancer types, we used TIMER as a comprehensive public database to systematically evaluate the clinical impact of different immune cells on different cancer types [30]. This database was used to analyze the IDH1 involvement in immune infiltration in prostate cancer.

### 4.7. Connectivity Map

Based on the gene expression map, we analyzed gene microarray chips for PRAD to obtain the IDH1 performance profile of possible drugs and then further screened the drugs with specificity and FDA approval through integration with bioinformatics [31]. The potential drugs were then evaluated using a connectivity map based on the genetic performance differences generated by different drug treatment of cell lines in the 6,100-slice Drug Connectivity Map.

### 4.8. RNA Extraction and Real-Time PCR

RNA was extracted using the EasyPrep Total RNA Kit (BIOTOOLS Co., Ltd., Taipei, Taiwan.) as previously described [18]. cDNA was synthesized using a ToolScript MMLV RT kit (BIOTOOLS Co., Ltd.). qPCR was carried out using a StepOnePlusTM system (Applied Biosystems, CA, USA) with TOOLS 2X SYBR qPCR Mix (BIOTOOLS Co., Ltd.).

## 5. Conclusions

In conclusion, significantly elevated IDH1 expression was associated with the poor prognosis of PRAD. In addition, IDH1 may affect the tumor microenvironment and immune cell infiltration in PRAD. Therefore, IDH1 may serve as a meaningful diagnostic and prognostic biomarker and immune-related therapeutic target for PRAD. Further studies are needed to confirm these findings and to explore the mechanisms and immunomodulatory functions of IDH1 in PRAD. The present study identified positive associations between IDH1 and the immune response and provides a novel direction for further investigations of PRAD etiology.

## Data Availability

The data that support the findings of this study are available from the corresponding author upon reasonable request.

## References

[1] Siegel R. L., Miller K. D., Fuchs H. E., Jemal A. (2021). Cancer statistics, 2021. *CA: A Cancer Journal for Clinicians*.

[2] Rebbeck T. R., Devesa S. S., Chang B.-L. (2013). Global patterns of prostate cancer incidence, aggressiveness, and mortality in men of African descent. *Prostate Cancer*.

[3] Bostwick D. G., Burke H. B., Djakiew D. (2004). Human prostate cancer risk factors. *Cancer*.

[4] Ye J., Gu Y., Zhang F. (2017). IDH1 deficiency attenuates gluconeogenesis in mouse liver by impairing amino acid utilization. *Proceedings of the National Academy of Sciences of the United States of America*.

[5] Lu C., Venneti S., Akalin A. (2013). Induction of sarcomas by mutant IDH2. *Genes & Development*.

[6] Yan H., Parsons D. W., Jin G. (2009). IDH1 and IDH2 mutations in gliomas. *The New England Journal of Medicine*.

[7] Figueroa M. E., Abdel-Wahab O., Lu C. (2010). Leukemic IDH1 and IDH2 mutations result in a hypermethylation phenotype, disrupt TET2 function, and impair hematopoietic differentiation. *Cancer Cell*.

[8] Dang L., White D. W., Gross S. (2009). Cancer-associated IDH1 mutations produce 2-hydroxyglutarate. *Nature*.

[9] Mateo J., McKay R., Abida W. (2020). Accelerating precision medicine in metastatic prostate cancer. *Naturaliste Canadien*.

[10] Fay E. K., Graff J. N. (2020). Immunotherapy in prostate cancer. *Cancers*.

[11] Subramanian A., Narayan R., Corsello S. M. (2017). A next generation connectivity map: L1000 platform and the first 1,000,000 profiles. *Cell*.

[12] Tung S.-Y., Pu Y.-S., Huang C.-Y. (2018). Outcomes of prostate atypical small acinar proliferation and high-grade prostate intraepithelial neoplasm patients. *Urological Science*.

[13] Lu-Yao G. L., Yao S.-L. (1997). Population-based study of long-term survival in patients with clinically localised prostate cancer. *The Lancet*.

[14] Wilt T. J., Brawer M. K., Jones K. M. (2012). Radical prostatectomy versus observation for localized prostate cancer. *The New England Journal of Medicine*.

[15] Wilt T. J., Brawer M. K., Jones K. M. (2012). Radical prostatectomy versus observation for localized prostate cancer. *New England Journal of Medicine*.

[16] Kelly S. P., Anderson W. F., Rosenberg P. S., Cook M. B. (2018). Past, current, and future incidence rates and burden of metastatic prostate cancer in the United States. *European Urology Focus*.

[17] Singh D., Febbo P. G., Ross K. (2002). Gene expression correlates of clinical prostate cancer behavior. *Cancer Cell*.

[18] Mortensen M. M., Høyer S., Lynnerup A.-S. (2015). Expression profiling of prostate cancer tissue delineates genes associated with recurrence after prostatectomy. *Scientific Reports*.

[19] Amary M. F., Bacsi K., Maggiani F. (2011). IDH1 and IDH2 mutations are frequent events in central chondrosarcoma and central and periosteal chondromas but not in other mesenchymal tumours. *The Journal of Pathology*.

[20] Hinsch H., Brolund M., Hube-Magg C. (2018). Immunohistochemically detected IDH1(R132H) mutation is rare and mostly heterogeneous in prostate cancer. *World Journal of Urology*.

[21] Hinsch A., Brolund M., Hube-Magg C. (2018). Immunohistochemically detected IDH1(R132H) mutation is rare and mostly heterogeneous in prostate cancer. *World Journal of Urology*.

[22] Kim S. H., Kim H., Lee J. H., Park J. W. (2019). Oxalomalate suppresses metastatic melanoma through IDH-targeted stress response to ROS. *Free Radical Research*.

[23] Yang J. H., Park J. W. (2003). Oxalomalate, a competitive inhibitor of NADP+-dependent isocitrate dehydrogenase, enhances lipid peroxidation-mediated oxidative damage in U937 cells. *Archives of Biochemistry and Biophysics*.

[24] Irace C., Esposito G., Maffettone C. (2007). Oxalomalate affects the inducible nitric oxide synthase expression and activity. *Life Sciences*.

[25] Moreira D. M., Howard L. E., Sourbeer K. N. (2017). Predicting time from metastasis to overall survival in castration-resistant prostate cancer: results from SEARCH. *Clinical Genitourinary Cancer*.

[26] Li J. Y., Li C. J., Lin L. T., Tsui K. H. (2020). Multi-omics analysis identifying key biomarkers in ovarian cancer. *Cancer Control: Journal of the Moffitt Cancer Center*.

[27] Tang Z., Kang B., Li C., Chen T., Zhang Z. (2019). GEPIA2: an enhanced web server for large-scale expression profiling and interactive analysis. *Nucleic Acids Research*.

[28] Zhang B., Kirov S., Snoddy J. (2005). WebGestalt: an integrated system for exploring gene sets in various biological contexts. *Nucleic Acids Research*.

[29] Vasaikar S. V., Straub P., Wang J., Zhang B. (2018). LinkedOmics: analyzing multi-omics data within and across 32 cancer types. *Nucleic Acids Research*.

[30] Li C. J., Chiu Y. H., Chang C., Chang Y. I., Sheu J. J., Chiang A. J. (2021). Acetyl coenzyme a synthase 2 acts as a prognostic biomarker associated with immune infiltration in cervical squamous cell carcinoma. *Cancers*.

[31] Li C. J., Lin L. T., Chu P. Y. (2021). Identification of novel biomarkers and candidate drug in ovarian cancer. *Journal of Personalized Medicine*.

